# Conclusiveness of Cochrane Reviews on Nursing Interventions for Patients with Cancer: A Systematic Analysis

**DOI:** 10.31662/jmaj.2023-0181

**Published:** 2024-04-01

**Authors:** Jun Kako, Masamitsu Kobayashi, Kohei Kajiwara, Yoshiyasu Ito, Michihiro Tsubaki, Takahiro Kakeda

**Affiliations:** 1 Graduate School of Medicine, Mie University, Tsu, Japan; 2 Graduate of Nursing Science, St. Luke’s International University, Tokyo, Japan; 3 Faculty of Nursing, Japanese Red Cross Kyushu International College of Nursing, Munakata, Japan; 4 College of Nursing Art and Science, University of Hyogo, Akashi, Japan; 5 School of Nursing, Kitasato University, Sagamihara, Japan; 6 Faculty of Nursing, Kawasaki City College of Nursing, Kawasaki, Japan

**Keywords:** Cochrane reviews, conclusiveness, nursing interventions, oncology nursing, systematic search

## Abstract

**Background:**

This study aimed to assess the conclusiveness of Cochrane Reviews (CRs) in oncology nursing.

**Methods:**

We searched systematically for all CRs published in the Cochrane Library in the oncology nursing field between January 2014 and April 2023. We analyzed the difference between conclusive and inconclusive outcomes using the χ^2^ and Mann-Whitney *U*-tests and identified 430 articles. However, we excluded 385 articles after reviewing their titles and abstracts. We assessed 45 full-text articles for eligibility and identified 32 articles. Of the 32 articles, we extracted 19 interventions.

**Results:**

The overall outcomes were 182 cases, with 51.6% (n = 94) and 48.4% (n = 88) demonstrating conclusiveness and inconclusiveness, respectively. Regarding conclusiveness, 28.0% (n = 51) and 23.6% (n = 43) reported that the studied interventions were effective and ineffective, respectively. We found that studies on interventions related to physical activity and yoga had significantly high rates of conclusive. Compared with inconclusiveness outcomes, conclusive outcomes involved significantly more studies (*p* < 0.001) and patients (*p* < 0.001).

**Conclusions:**

Ultimately, these findings reveal that in the oncology nursing field, only 51% of the main outcomes of each nursing intervention in CRs were conclusive.

## Introduction

Cochrane is an international network headquartered in the United Kingdom with members and supporters from over 190 countries worldwide (https://www.cochrane.org/). Its vision focuses on improving worldwide health and normalizing evidence-based decision-making in health care. It produces trusted evidence, advocates for evidence, informs health and care decisions, and provides high-quality information to help end users receive evidence-based medicine. Grimshaw notably affirmed the organization’s work: “The Cochrane Library is the best single resource for methodologic research and for developing the science of meta-epidemiology ^(1)^.”

Consequently, Cochrane Reviews (CRs) have become the international standard for evidence-based medicine. More specifically, Cochrane is known for producing high-quality systematic reviews based on randomized controlled trials (RCTs). However, not all the information provided by Cochrane achieves the goals of a CR. Indeed, not all CRs are conclusive, which is defined as a situation where one intervention is superior to the other or when both interventions are equivalent. Conversely, inconclusiveness is characterized by insufficient data ^(2)^. According to previous studies, 45%-80% of CRs are conclusive ^(2), (3), (4), (5), (6), (7)^. However, even when conclusiveness is achieved, additional high-quality research is often required.

This study explored the clinical conclusiveness of CRs by focusing on their frequency in existing research on oncology nurses. We focused on this field because oncology nursing is increasingly playing an essential role given the growing global burden of cancer today ^(8)^. The Oncology Nursing Society states that the roles of oncology nurses are crucial in the lives of those requiring care in the present healthcare landscape, which is “developing in ways that position us to be stronger advocates than ever before ^(9)^.” Oncology nurses deliver care, conduct clinical trials, and advance evidence-based research.

Evidence for nursing interventions in the field of cancer has accumulated; for example, recent clinical practice guidelines for dyspnea among patients with cancer focus on nonpharmacological interventions offered by nurses, which are described as first-line treatments for dyspnea ^(10), (11)^. Notably, nurses can provide many interventions described in these clinical practice guidelines. However, CRs in oncology nursing have not been summarized systematically. As such, the nursing interventions reported in CRs and the extent to which each nursing intervention is considered conclusive remain unclear. Investigating recent CRs in the oncology nursing field may help identify useful directions for future research in this field. Therefore, this study established the clinical conclusiveness of CRs in oncology nursing.

## Materials and Methods

### Search strategy and selection criteria

We searched systematically for all CRs published in the Cochrane Library between January 2014 and April 2023 in the oncology nursing field. (The search strategy is available upon request from the corresponding author.) We included reviews that assessed the effects of oncology nursing, defined as any type of intervention provided by nurses. Many nurse-led intervention studies often exclude details on the interventionists ^(12)^. Therefore, if the care provider was not stated, the researchers, including oncology nurses, discussed whether nurses could provide the kind of care in question in their daily clinical practice. If the researchers agreed that nurses could provide this type of care, it was identified as a nursing intervention. Complex interventions were also included if the nurse was included as a care provider. Outdated versions, withdrawn manuscripts, and protocol reviews were excluded.

### Study selection process

Two authors (J.K. and M.K.) independently assessed the titles and abstracts of the studies, followed by full-text screening against the eligibility criteria. We resolved disagreements through consensus among authors or discussion with another author (K.K., Y.I., M.T., and T.K.). We recorded the selection process and created a PRISMA flow diagram.

### Data extraction

The same authors (J.K. and M.K.) independently extracted data from all CRs that met the criteria using a standard form. They extracted the first author, year of publication, type of nursing intervention, primary outcome, conclusiveness or inconclusiveness, and number of RCTs from each CR. Disagreements were resolved through discussions.

The conclusiveness or inconclusiveness of the reviews was assessed as follows ^(7), (13)^. A review was deemed conclusive if (1) one intervention was more effective than the other and (2) the interventions were equally effective. Conversely, it was assessed as inconclusive if there was no decision because the quality of the study and data were inadequate or existing RCTs were outdated. Importantly, as in previous studies ^(2), (3), (4), (5)^ only the main outcome of each review (rather than the secondary outcomes) was considered.

### Statistical analysis

We collected descriptive statistics on “conclusive” and “inconclusive” outcomes and analyzed the difference between these outcomes using the χ^2^ test and the Mann-Whitney *U*-test. A *p*-value of <0.05 was considered significant ^(2), (3), (4), (5), (6), (7), (13)^. We used EZR (Saitama Medical Center, Jichi Medical University, Saitama, Japan) and R (The R Foundation for Statistical Computing, Vienna, Austria) ^(14)^ for statistical analyses.

## Results

### Search results

**Figure 1.**
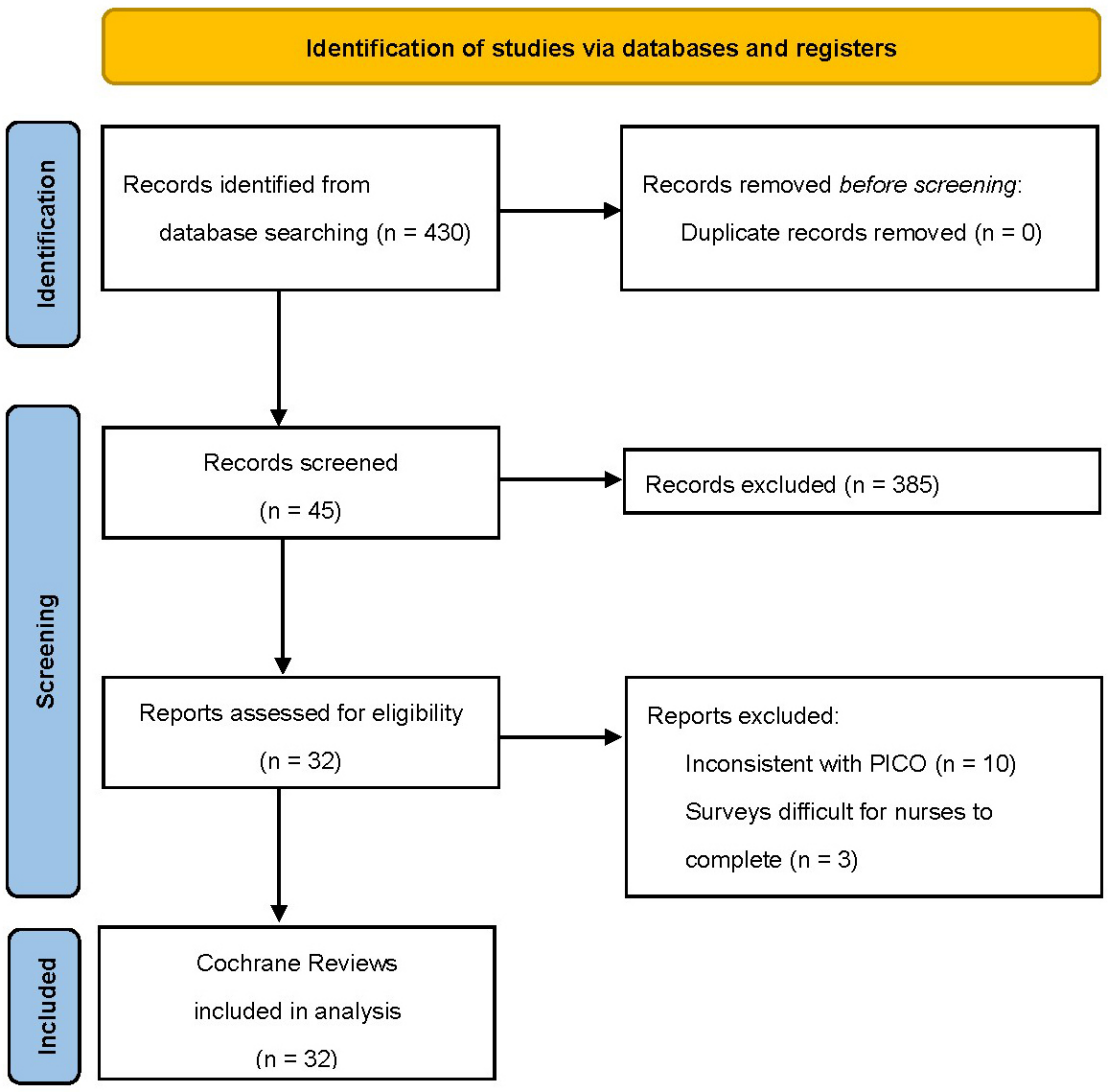
PRISMA flow diagram; PICO, patient or problem, intervention, comparison, and outcome.

Figure 1 shows the literature screening process and results. We identified 430 articles; however, we excluded 385 articles after reviewing their titles and abstracts. We assessed 45 full-text articles for eligibility and excluded 13 articles because of either inconsistency with patient or problem, intervention, comparison, and outcome (n = 10) or participant nurses finding it difficult to complete the study surveys (n = 3).

### Nursing intervention group

From the 32 articles, 19 interventions were extracted: aerobic physical exercise ^(15)^, cognitive behavioral therapy ^(16), (17), (18)^, compensatory strategy training ^(17)^, dietary interventions ^(19), (20)^, early palliative care ^(21), (22)^, education ^(23), (24), (25), (26), (27)^, exercise ^(23), (26), (28), (29), (30), (31), (32), (33), (34)^, manual lymph drainage ^(23)^, meditation ^(17)^, mindfulness ^(35)^, multidisciplinary interventions ^(27)^, multimodal interventions ^(28), (36)^, multimodal prehabilitation ^(37)^, oral cryotherapy ^(38)^, physical activity intervention ^(39), (40)^, physical interventions ^(27)^, psychosocial interventions ^(41), (42)^, telephone interventions ^(43)^, and yoga ^(44), (45)^.

### Conclusiveness of CRs

Table 1 shows the nursing intervention groups extracted from the CRs, the number of main outcomes adopted for each nursing intervention, and the percentage of outcomes in conclusive or inconclusive reviews. The nursing interventions with the highest number of main outcomes were physical activity (n = 40), exercise (n = 27), yoga (n = 18), education (n = 17), and psychosocial interventions (n = 13). Multidisciplinary (n = 1) and physical (n = 1) interventions had the lowest number of main outcomes.

**Table 1.** Conclusiveness of CRs in the Nursing Intervention Group.

Nursing intervention group	Conclusiveness (%)		Inconclusiveness (%)	*p*
	Effective	No difference		
Aerobic physical exercise (n = 2)	0	50.0	50.0	1
Cognitive behavioral therapy (n = 7)	57.1	0	42.9	1
Compensatory strategy training (n = 2)	0	0	100	0.232
Dietary interventions (n = 6)	0	0	100	0.0117
Early palliative care (n = 11)	36.4	27.3	36.4	0.539
Education (n = 17)	23.5	5.9	70.6	0.0737
Exercise (n = 27)	22.2	22.2	55.6	0.532
Manual lymph drainage (n = 3)	100	0	0	0.247
Meditation (n = 3)	0	0	100	0.111
Mindfulness (n = 2)	0	0	100	0.232
Multidisciplinary interventions (n = 1)	100	0	0	1
Multimodal interventions (n = 6)	33.3	33.3	33.3	0.683
Multimodal prehabilitation (n = 4)	0.0	75.0	25.0	0.622
Oral cryotherapy (n = 10)	40.0	20.0	40.0	0.748
Physical activity interventions (n = 40)	42.5	40.0	17.5	<0.001
Physical interventions (n = 1)	0	0	100	0.486
Psychosocial interventions (n = 13)	0	7.7	92.3	<0.001
Telephone interventions (n = 9)	0	0	100	0.0032
Yoga (n = 18)	33.3	44.4	22.2	0.0246

n, number adopted as the main outcome in the included CRs CRs, Cochrane reviews

In total, we identified 182 outcomes. Among these outcomes, 51.6% (n = 94) and 48.4% (n = 88) were conclusive and inconclusive, respectively. Regarding conclusive outcomes, 28.0% (n = 51) and 23.6% (n = 43) involved an effective and an ineffective intervention, respectively. We compared the outcomes of each nursing intervention for conclusiveness and inconclusiveness using the χ^2^ test; the results showed that interventions related to physical activity and yoga had significantly high rates of conclusiveness.

Table 2 illustrates the characteristics of the CRs of RCTs in nursing interventions. Compared with inconclusive outcomes, conclusive outcomes involved significantly more studies (mean 4.34 times higher, *p* < 0.001) and patients (*p* < 0.001).

**Table 2.** Study Characteristics of CRs of RCTs in Nursing Interventions.

	Conclusiveness outcomes		*p*
	Yes (n = 94)	No (n = 88)	
Studies enrolled	7.56 ± 6.10 (2-27)	1.74 ± 2.10 (0-9)	<0.001
Patients enrolled	721.7 ± 647.7 (106-3,321)	318.6 ± 567.6 (0-3,107)	<0.001

Data are expressed as mean ± standard deviation (range). CRs, Cochrane reviews; RCTs, randomized controlled trials

## Discussion

Previous analyses of examined interventions overseen by oncology nurses indicate that most existing studies have low reporting ^(12)^ and methodological quality ^(46)^. Therefore, enhancing the quality of clinical research on oncology nursing field remains challenging. In response, this study investigated recent CRs in the oncology nursing field to uncover the frequency of obtaining conclusive results for different nursing interventions. Because we evaluated the conclusiveness or inconclusiveness of the main outcomes of each nursing intervention, our results may not be comparable with those of previous studies that evaluated each CR ^(2), (7)^.

Broadly, we found that conclusive reviews accounted for 51.6% of CRs; accordingly, many studies on nursing interventions were inconclusive. Notably, although CRs often lead to definitive clinical recommendations, CRs in oncology nursing do not always provide well-informed conclusions about the outcomes of each nursing intervention. Regarding the conclusive outcomes, 28.0% and 23.6% reported effective interventions and no changes after the intervention, respectively. We also found that studies on interventions related to physical activity and yoga had significantly high rates of conclusive. Furthermore, this study demonstrated that the number of participating patients and RCTs affects the ability of a CR to conclude. This result was consistent with that of previous studies ^(2), (3), (4), (5), (6), (7)^. However, the number of included RCTs was generally small―approximately 7.6 and 1.7 RCTs were included in the conclusive and inconclusive studies, respectively. This result indicates that nursing interventions were inconsistently evaluated for the same outcomes.

Future studies should refer to existing research policies that recommend standardizing tools for measuring outcomes to enable comparisons between studies and study integration. For example, in Japan, the Supportive and Palliative Care Research Policy was developed to provide guidelines for outcome evaluations in intervention studies ^(47), (48), (49)^. The research policy for dyspnea in patients with cancer recommends that the intensity of dyspnea should be measured using a patient-reported outcome scale (e.g., numerical rating scale, visual analog scale, the modified Borg scale, cancer dyspnea scale, and multidimensional dyspnea profile), which should be adopted as the main outcome in clinical research on dyspnea associated with cancer treatment ^(48)^.

This study had some limitations. First, we excluded systematic reviews published in other journals or databases because non-CRs often have different levels of methodological quality. Second, not all the nursing care extracted in this study is routinely provided by nurses worldwide because the scope of care provided by nurses varies from country to country. Third, the role of the oncology nurse is broadly defined because there is no worldwide collective term for oncology nursing ^(8)^. Therefore, the results of this study do not define the role of the oncology nurse. Fourth, the number of RCTs for each outcome was limited because we evaluated the conclusiveness of each main outcome of each nursing intervention in CRs. These potential biases were identified during the analysis and could have impacted the study results. Finally, this study counted outcomes that were assessed using RCTs included in the CRs; therefore, there is an overlap of RCTs across the outcomes, which may have biased the results.

## Conclusions

In the oncology nursing field, only 51% of the main outcomes of each nursing intervention in CRs were conclusive. Compared with inconclusive outcomes, conclusive outcomes were more popular in reviews that involved significantly higher numbers of studies and patients. However, this study was notably limited in that it included a relatively small number of RCTs; therefore, it is necessary to conduct RCTs using high-quality and consistent evaluation tools in the future.

## Article Information

### Conflicts of Interest

None

### Sources of Funding

This work was supported by JSPS KAKENHI grant number 21H03236. The funders played no role in the study design, data collection and analysis, publication decision, or manuscript preparation.

### Acknowledgement

We thank Editage (www.editage.com) for their assistance with English language editing.

### Author Contributions

All authors (J.K., M.K., K.K., Y.I., M.T., and T.K.) contributed to the preparation, drafting, and editing of Cochrane Reviews. J.K. and M.K. conceived the research idea, after which discussions with other authors (K.K., Y.I., M.T., and T.K.), who contributed to the finalization of the research idea, were held. J.K. and M.K. developed data extraction and systematic database search strategies. All authors contributed to the preparation and editing of the manuscript and have read and approved the final version.

### Data Availability Statement

All relevant data have been included in this study.

### Patient Consent for Publication

Not applicable.

### Ethics Approval

Not applicable.
